# Influencing Factors of Patients’ Trust in Nurses During the COVID-19 Pandemic: A Mixed-Methods Study

**DOI:** 10.1017/dmp.2022.262

**Published:** 2022-11-03

**Authors:** Xiaolan Tang, Junhua Lu, Zhihui Chen, Chao Liu, Xue Jiang, Mei Ning

**Affiliations:** 1 College of Nursing, Mudanjiang Medical University, Mudanjiang, Heilongjiang Province, China; 2 Department of Emergency, Hongqi Hospital Affiliated to Mudanjiang Medical University, Mudanjiang, Heilongjiang Province, China; 3 Department of General Surgery, Hongqi Hospital Affiliated to Mudanjiang Medical University, Mudanjiang, Heilongjiang Province, China

**Keywords:** COVID-19, mixed research, nurse–patient trust

## Abstract

**Objective:**

This study aimed to investigate the nurse-patient trust among in-patients in the context of the coronavirus disease (COVID-19) epidemic; it further analyzed the related influencing factors, which will provide a theoretical basis for developing corresponding measures.

**Methods:**

This study employed a mixed-method design and analyzed 149 patients at the Hongqi Hospital, affiliated with Mudanjiang Medical University, from December 2020 to February 2021. Quantitative analysis was carried out using the “Nurse Patient Trust Scale,” and qualitative analysis was performed using a semi-structured interview with in-patients.

**Results:**

The average score on the scale was 46.65 ± 2.83, and the scores of the 2 dimensions were: 23.24 ± 1.51 for ability and peace of mind, and 23.32 ± 1.53 for attitude and care. According to the interview data, the factors included 3 aspects: a comfortable hospital environment and humane management measures; the nurse’s own competence; and effective communication with patients.

**Conclusion:**

During the COVID-19 epidemic, there are still many factors affecting patients’ trust in nurses that can be addressed by taking different measures. All these factors must be considered by the relevant managers and clinical nursing staff to maintain a better nurse-patient trust relationship.

Trust is the basis of cooperation; it means trusting the other person without fear, hesitation, doubt, observing ethical norms, and handling conflicts with dignity and goodwill without harming others.^1^ Good nurse-patient relationships are key to treatment progression.^2^ Trust as an essential part of nursing practice plays a vital role in building a good nurse-patient relationship.^3^ Especially in health care, a good nurse-patient relationship would help promote the treatment of patients, create a harmonious medical atmosphere, and enhance the nurses’ motivation.^4,5^ However, when a patient is ill or experiencing other risk factors, the trust between nurse and patient is very fragile and, once lost, is not easily restored, the consequences of which will directly impact nursing practice, education, and management.^3^ It is therefore important to establish a good nurse-patient trust relationship. Multiple factors influence the nurse-patient relationship. One study showed that the availability and accessibility of nurses and patients’ emotional and physical safety tend to influence patients’ trust in nurses.^6^ Also, the trust between nurses and patients is susceptible to environmental influences, including whether the nurse has enough time to communicate with patients to inform them about their illnesses, the effects of the medicines, and the timing of the review.^7^

To some extent, the epidemic outbreak raised nurses’ social position. In this pandemic, nurses have been on the front line, providing various important services to patients, families, and communities. Their contributions were acknowledged by all, especially by patients in the hospital.^8^ However, the severe prevention and control management of hospitals led to difficulties for patients’ family members in accompanying the patient to the hospital. This increased the psychological burden on in-patients and, somewhat, also affected the nurse-patient trust.^9–11^

Trust in nurses is seen as a very important factor in nurse-patient interaction.^12^ However, few studies have been conducted to investigate the factors that influence patients’ trust in the relationship with nurses during an epidemic. Therefore, the purpose of this study is to investigate the factors affecting in-patients’ trust in nurses during the COVID-19 pandemic and to provide practical coping strategies for hospital managers.

## Patients and Methods

### Study Design and Setting

This research used a mixed-method design, combining quantitative and qualitative aspects from December 2020 to February 2021 at Hongqi Hospital, Mudanjiang Medical University, China. For the quantitative study, we used the Nurse-Patient Trust Scale (NPTS), which was prepared by the Japanese scholar Gangguhuizi in 1995, and the Chinese scholars Zhao et al. Sinicized and modified it according to the Chinese context in 2018; Cronbach’s α of the scale was more than 0.8. We used the scale of NPTS developed by the Chinese scholars Zhao et al.^13^ For the qualitative study, 4 points total were included through a semi-structured interview outline of our research team’s own design.

### Sample Size

The sample size for quantitative studies is mostly based on the requirements of statistical variable analysis. The number of samples should be at least 5–10 times of the number of variables.^14^ According to this study, the number of questionnaire items should be 12, so the calculated sample size N = 12 × 10 = 120. Then, we added 20% to the sample size for non-response or for selection bias, thus, making the sample size 144. Finally, 149 patients were included for further analysis. For the qualitative study, 10 patients were finally included until the interview data were saturated.

### Study Participants and Recruitment

#### Quantitative analysis

A total of 149 patients hospitalized in Hongqi hospital affiliated with Mudanjiang Medical University, China, from December 2020 to February 2021, selected by convenient sampling, were investigated by the questionnaire (NPTS).

##### Inclusion criteria

(1) Age ≥ 18 years; (2) Patients admitted ≥ 3 days; (3) Patients with clear consciousness and independent judgment ability; (4) Patients who signed informed consent and voluntarily participated in this survey; and (5) Patients who are not infected with the new coronavirus.

##### Exclusion criteria

(1) Patients with consciousness, speech, mental, neurological, and other physical diseases; (2) Critically ill patients; (3) Patients who cannot actively cooperate in the study; and (4) Re-admitted patients who have been investigated.

#### Qualitative analysis

After collecting quantitative data, patients above and below the mean scores (mean scores = 46.65) were divided into 2 groups. Ten patients from each group were then selected by a nurse in our department. The semi-structured interviews were conducted with the sampled patients until the required information was collected (if a patient was discharged, the next patient was interviewed) until no new items emerged. Finally, 10 patients were interviewed.

### Data Collection Tools

A quantitative study was conducted using the NPTS. For the qualitative study, in-depth interviews were conducted with the participants, based on the analysis of the quantitative study, using semi-structured interview materials designed by the research team.

#### General data questionnaire

The researchers designed the questionnaire, including gender, age, residence, sleep, hospitalization time, expenses, marriage, education level, occupation, medical insurance, and income.

#### Nurse-Patient Trust Scale (NPTS)

This study adopted the NPTS after cultural adaptation by Zhao et al., related to the Chinese national context.^13^ The scale was revised to include 12 entries with 2 dimensions, 6 entries for attitude and caring (3, 4, 5, 6, 11, 12), and 6 entries for competence and reassurance (1, 2, 7, 8, 9, 10). The 4-point Likert scale (disagree = 1, partially agree = 2, basically agree = 3, fully agree = 4) was utilized, with no reverse scoring entries, and patients had to choose the appropriate content based on their feelings. The total score ranged from 12 to 48 points, with higher scores indicating higher nurse–patient trust. According to the results of this study, Cronbach’s α and half coefficients for the total scale and the 2 dimensions were above 0.86, indicating good reliability and validity.

#### Interview outline of patients’ trust in nursing

The interview outline, designed by researchers, revolves around the experiences and feelings of patients in contact with nurses in the context of the COVID-19 epidemic. The interview questions were the 4 that followed: (1) Do you trust nurses or nursing operations? (2) Have you ever been touched or seen anything in a way that affects your trust in nursing? (3) How do you handle the relationship with nurses? (4) Is there any particular reason for hospitalization that affects your relationship with nurses?

### Data Collection

#### Quantitative data

The investigators received uniform training involving how to communicate with patients, motivate them to fill in questionnaires effectively, and ensure the authenticity of the questionnaires to prevent the surveyors from influencing the study. The respondents were introduced to the purpose, precautions, and confidentiality prior to the survey. After the patients’ consent was obtained, questionnaires were distributed and collected after completion. The collected data were numbered and counted. About 160 questionnaires were distributed, and 149 were collected, with an overall response rate of 93.1%.

#### Qualitative data

Before the interview, patients were informed of the purpose of the interview. During the interview, the recording and data were kept confidential and replaced by numbers. The interview was conducted for about 30–40 minutes. Ten patients were interviewed, and the data were collated and recorded within 24 hours.

### Data Analysis

#### Quantitative data

SPSS 26.0 software (IBM Inc., Chicago, IL, USA) was used for the statistical analysis. General data of the research objects were presented using frequency. The measurement data were expressed as mean and standard deviation, and the influencing factors were tested by T-test/F-test, with a significant level of α = 0.05.

#### Qualitative data

The descriptive qualitative study was used to analyze the interview data by content analysis.^15^ NVivo12 (US) was used to organize materials and memos in time, concepts and themes were gradually formed, and the preliminary theme framework was subsequently sorted out. In addition, expressions relevant to this study were refined, coded sentence by sentence, and continually compared and categorized with existing thematic frameworks until no new themes and subthemes emerged.

## Results

### Quantitative Research

The data of 149 patients were collected. There were 85 (57%) males and 64 (43%) females. The vast majority of the participants (85.9%) were married, and the rest were unmarried, divorced, or widowed. In terms of education, 38.9% received a senior high school education or above. Among respondents, 8.7% had a monthly household income of $5000 CNY or more (Table 1).

**Table 1.** Sample characteristics (n = 149)

Variable	n	%
Gender	85	57
Male		
Female	64	43
Sleep quality	49	32.9
Abnormal		
Normal	100	67.1
Marriage	21	14.1
Other		
Married	128	85.9
Occupation	97	65.1
Retired		
On the job	52	34.9
Medical insurance	13	8.7
Self-paying		
Medical insurance	136	91.3
Age (years)	16	10.7
≤ 40		
40-60	59	39.6
60-80	65	43.6
> 80	9	6
Average monthly household income		
< 2000	35	23.5
2001-3000	51	34.2
3001-4000	36	24.2
4001-5000	14	9.4
> 5000	13	8.7
Place of residence		
Urban area	74	49.7
Rural area	75	50.3
Hospital stay (d)		
≤ 10	102	68.5
11-20	35	23.5
21-30	8	5.4
31-40	2	1.3
> 40	2	1.3
Hospitalization expenses (CNY)		
≤ 5000	24	16.1
5001-10 000	57	38.3
10 001-20 000	37	24.8
20 001-30 000	16	10.7
> 30 000	15	10.1
Education		
Primary school and below	38	25.5
Junior school	53	35.6
Senior high school	27	18.1
University or above	31	20.8

### Current Situation of Nurse-Patient Trust

The total score of the questionnaire was 46.64 ± 2.83 points. The top 3 scores were: “When I have an emergency, the nurse can handle it correctly (3.93 ± 0.26),” “After listening to the nurse’s explanation, I feel much better (3.93 ± 0.26),” and “The nurse encourages me to overcome the disease (3.92 ± 0.30).” The score of each item is shown in Table 2. The last 3 items are: “Nurses’ knowledge is comprehensive (3.8 ± 0.41),” “Nurses consider and respect my ideas (3.84 ± 0.40),” and “I trust nurses’ guidance (3.85 ± 0.39).”

**Table 2.** Scores of items in nurse–patient trust questionnaire

Dimension	Item	Score	Rank
Ability and peace of mind	*What nurses can or cannot do can be explained clearly. Nurses are consistent with their words and deeds.*	3.91 ± 0.28 3.91 ± 0.28	4 4
	*When I have an emergency, the nurse can handle it correctly.*	3.93 ± 0.26	1
	*I trust the guidance of nurses.*	3.85 ± 0.39	10
	*Nurses have comprehensive knowledge.*	3.81 ± 0.41	12
	*I can rest assured that the nurse cares for me.*	3.91 ± 0.29	6
Attitude and care	*Nurses consider and respect my ideas.*	3.84 ± 0.40	11
	*Talking with nurses enhances confidence for the future.*	3.89 ± 0.31	7
	*The nurses encouraged me to overcome the disease.*	3.92 ± 0.30	3
	*After listening to the nurse’s explanation, I feel much better.*	3.93 ± 0.26	1
	*The nurse can listen to me patiently.*	3.87 ± 0.35	9
	*The nurse speaks reasonably.*	3.89 ± 0.34	8

### Analysis of Influencing Factors in the Nurse-Patient Trust

The variables of sleep quality level were found to be significant (*P* < 0.05) (Table 3) in the univariate analysis. This suggests that during COVID-19, patients’ trust in nurses was influenced by their sleep status, with patients who had poor sleep having less trust in nurses.

**Table 3.** Analysis of nurse–patient trust

Variable	Average value ± standard deviation	t/f	*P*
Gender			
Male	46.62 ± 2.89	−0.136	0.892
Female	46.69 ± 2.78		
Sleep quality			
Abnormal	45.65 ± 3.74	−2.585	0.012
Normal	47.14 ± 2.11		
Marriage			
Other	46.1 ± 3.81	−0.97	0.334
Married	46.74 ± 2.65		
Occupation			
Retired	46.69 ± 2.8	0.233	0.816
On the job	46.58 ± 2.93		
Medical insurance			
Self-paying	46.31 ± 3.71	−0.456	0.649
Medical insurance	46.68 ± 2.75		
Age (years)			
≤ 40	46.00 ± 4.12		
40-60	46.85 ± 2.14	0.579	0.629
60-80	46.54 ± 3.16		
> 80	47.33 ± 1.32		
Average monthly household income			
≤2000	46.91 ± 2.03		
2001-3000	46.31 ± 3.57	1.087	0.365
3001-4000	46.92 ± 2.43		
4001-5000	47.5 ± 0.85		
> 5000	45.62 ± 3.64		
Place of residence			
Urban area	46.35 ± 3.27	−1.285	0.201
Rural area	46.95 ± 2.31		
Hospital stay (d)			
≤ 10	46.72 ± 2.73		
11-20	46.71 ± 2.71	1.519	0.2
21-30	46.38 ± 3.02		
31-40	42 ± 8.49		
> 40	48 ± 0		
Hospitalization expenses (CNY)			
≤ 5000	46.58 ± 3.01		
5001-10 000	46.60 ± 2.92	1.581	0.197
10 001-20 000	46.89 ± 2.49		
20 001-30 000	45.53 ± 3.73		
> 30 000	47.64 ± 1.34		
Education			
Primary school and below	46.11 ± 3.2		
Junior school	47.43 ± 1.42	2.259	0.084
Senior high school	46.48 ± 2.98		
University or above	46.13 ± 3.73		

### Qualitative Research

The general demographic information of the interviewees is shown in Table 4. The influencing factors of the nurse-patient relationship trust are summarized into 2 aspects: the factors that are not conducive to the nurse-patient relationship trust and the factors that are conducive to the nurse-patient relationship trust. Further, these aspects were analyzed from the patient, nurse, and social background perspectives: comfortable hospital environment and humane management measures; nurses’ own competence; effective communication with patients.

**Table 4.** General demographic data of the interviewees (n = 10)

No	Gender	Age at interview	Education level	Place of residence	Length of stay (day)	Medical insurance	Personal income (CNY)	Score of confidence degree
P1	Male	49	Junior school	Rural	3	Medical insurance	≥ 6000	47
P2	Female	30	Junior school	Rural	16	Self-paying	≥ 6000	48
P3	Male	74	Primary school and below	Urban	10	Medical insurance	2001- 3000	48
P4	Female	53	Senior high school	Urban	3	Medical insurance	≤ 2000	48
P5	Male	75	University	Urban	6	Medical insurance	4001- 5000	48
P6	Female	64	University	Urban	9	Medical insurance	2001- 3000	35
P7	Male	43	Junior school	Rural	13	Medical insurance	3001- 4000	48
P8	Male	69	Primary school and below	Rural	3	Medical insurance	2001- 3000	48
P9	Male	62	Senior high school	Rural	10	Medical insurance	2001- 3000	44
P10	Female	66	Junior school	Urban	6	Medical insurance	2001- 3000	48

### Data Analysis

#### Adverse factors

##### Patient factors

(1) Patients do not recognize the expertise of nurses—P3: “I just got admitted to the hospital with many questions, and I think the doctors are too busy, so I want to talk to the nurses, but I always feel that what the nurses say may not be very clear”; P3: “I don’t usually ask the nurse if I have a problem. Should I not ask the doctor about treatment? If the infusion does not work, then, of course, you should ask the nurse”; P7: “It is the nurse who gives the injections, but it is better to ask the doctor. Doctors have more experience than nurses.” (2) Patients have high expectations. Patients hope for a good outcome, but the variability of their condition leads to physical and mental exhaustion and emotional sensitivity—P4: “If the test results are good, I hope someone will let me know because I am prone to overthink, and I will be too worried and afraid to ask”; P8: “I spend a lot of money, but I think the curative effect is too slow, the disease repeats itself, my wound is not easy to heal, and now it is still expanding.”

##### Nurse factors

(1) Nurses are often reluctant or unable to interact much with patients because they are overwhelmed by their work in nursing—P6: “Nursing was not well versed in some knowledge of the disease, they do not explain things enough and may also care for many patients, like when I want to ask them a question, they can be called away by something else before they make it clear”; P8: “I didn’t know anything when I was first admitted; I was dazed and confused, I had to undergo a lot of tests and treatments, and the nurses gave me notes, some of which I probably did not understand the technicality, I felt confused about what I was doing, and the nurses did not provide a detailed briefing.” (2) In a university hospital, many nursing students will come here to learn clinical knowledge—P3: “I do not want trainee nurses to perform operations on me; they are just practicing.”

##### Social background factors

(1) Hospital prevention and control management restrict access to patients, especially their families—P5: “We need to do many tests before I have to be hospitalized. My wife and I are getting older, and we want our children to go to the hospital to take care of us, but they cannot enter or leave once they are in but have to go to work, so it’s not convenient.” (2) Hospitalization costs are relatively high—P1: “We need to live in an isolation room with higher costs, and some tests are now added. For example, accompanying relatives also need nucleic acid tests. We can only eat the food distributed by the hospital canteen, which increases the cost and is not convenient. The overall cost of hospitalization is higher than before.” (3) Lack of waiting rooms—P10: “There are too many people on the ward, which I think is not conducive to the management of epidemic prevention and control. With several patients in one room, the air is not good, and masks cannot fully achieve the isolation effect. The greatest worry is that we will all be quarantined if there are such patients or suspected patients in the hospital.”

#### Favorable aspects

##### Patient factors

(1) Patients have empathy and can understand the difficulties of nursing workers from a nursing point of view—P1: “There are few nurses on the ward that need to take care of patients and the families of the patients. Moreover, they not only need to measure our temperature frequently but also help us pick up and deliver our daily necessities. I heard that some nurses still need to go to places with epidemic outbreaks, so it is necessary to understand their hard work”; P4: “It is not easy for a nurse. I am a patient with a stoma. I once burst into my pocket when I fell asleep at 1 am and filled the bed with stools. I was in a terrible mood at that time, and the night nurse saw my condition and took care of it immediately, changed my quilt, clothes, and made pockets, which helped me deal with it very well. They not only had good skills on their hands but also made me feel particularly warm”; P10: “Nurse are very busy every day. I usually do not ask them questions. I am afraid of increasing their workload. Anyway, I don’t understand medicine, but I trust them.”

##### Nurse factors

(1) Excellent professional skills: Nurses with professional skills are the basis to gain the trust of patients and enable them to better cooperate with treatment—P5: “The nurse in this department is exemplary. I am often hospitalized. The nurses here are diligent. Although they wear masks and I cannot see their faces, they often encourage me.” (2) Good professionalism: Exemplary professionalism can establish a harmonious atmosphere for medical treatment—P6: “I find that nurses are neatly dressed, energetic, meticulous, patient, and responsible every day. Every time they come in and out of the door, they keep their voice to a minimum”; P9: “My nurse asks me about my meals, sleep quality, and if I need help every day. I like to chat with her.”

##### Social background factors

(1) The hospital must strictly prevent and control the management system to avoid cross-infection in the hospital and ensure the safety of the patient—P1: “It is difficult to accompany family members now; there are many requirements, but, understandably, we have done an excellent job for our safety”; P2: “I saw nurses guarding the stairs”; P3: “The hospital is disinfected many times a day, and everyone has been tested. I am relieved.” (2) Media report on the outstanding contribution of nurses during the epidemic—P6: “I have been seeing nurse news lately, like the most beautiful nurses. I was touched by the nurses with whom I came in contact”; P7: “Nurse are very brave; I can see from TV news that many nurses went to Wuhan during the outbreak of the epidemic.”

## Discussion

The results of the NPTS showed that patients’ overall trust in nursing care was high at 46.64 ± 2.83 points. The results of the combined interviews indicated that various favorable factors had a positive effect on maintaining a good nurse-patient relationship. This may be because the value of nurses in the epidemic was highlighted through meticulous nursing measures, which greatly met the humanistic needs of patients during their hospitalization and promoted their affirmation of nursing care. Moreover, positive social media coverage contributed to a certain extent to patients’ understanding of nursing care, their trust in nursing workers, and their willingness to cooperate with treatment. However, there are still some factors that need our attention, including the patient’s sleep, the effectiveness of treatment of their own illness, the social context of the epidemic, and the nurses’ expertise. Three nursing measures are proposed to address these factors, including a comfortable hospital environment and humane management measures, the nurses’ own competence, and effective communication with patients.

### Comfortable Hospital Environment and Humane Management Measures

Previous research showed that there is a direct relationship between the patient’s sleep status and the level of nurse–patient trust, with environmental factors recognized as the most influencing.^16^ This may have been due to the lack of sleep for patients and the special in-patient environment of the hospital during the COVID-19 pandemic, whose complex admission procedures and strict hospital management processes further affected patients’ mental health status, leading to dissatisfaction with care and distrust of nurses.^17^ Meanwhile, some interviews showed that patients’ distrust of the hospital also led to patients’ distrust of their nursing work. During the COVID-19 pandemic, hospitals, as special places, are subject to strict requirements in different countries in order to mitigate the infectious disease hazards, protect health, and prevent the viral spread.^18,19^ Strict management may, to some extent, lead to cumbersome hospitalization, restrictions on prevention and control management, and increased costs, all of which can affect patients’ trust in their care.^20^

Even considering the need to adopt preventive and control measures in hospitals, health care professionals can create as comfortable an environment as possible for patients to sleep well while taking targeted measures for hospital management to ensure the overall safety as far as possible and reduce difficulties in admitting patients—for example, recruiting and training staff to help and guide patients through the admission process.^1^ It is also possible to classify hospitals into different levels according to their capacity so that they can receive and sort patients with different conditions.^21^ Establishment of a regional coordination unit for admissions is responsible for collecting daily data on the number of empty beds and reallocating the number of patients to facilitate rapid admissions wherever possible.^22^ We can also promote patient understanding by giving hospital management brochures to in-patients, by informing them about epidemic preparedness, and answering their questions in a timely manner. At the same time, the results of the study also showed a positive effect of news media for positive public guidance, but social media have the characteristics of complex information and fast dissemination and should be adopted in an appropriate way to promote the spreading of effective health knowledge.^23,24^ The mentioned methods were used to promote patients’ understanding and support of hospital work and nursing care and to increase the level of patients’ trust in nursing care.

### The Nurses’ Own Competence

Both the individual items of the NPTS questionnaire and the results of the interviews showed that patients felt that the nurses’ knowledge was incomplete. Similar results were reported by Zhang et al. They used the Newcastle Satisfaction with Nursing Care Scale, which is different from this study’s scale. Still, the survey population was the same as this study with Chinese patients.^25^ As people’s quality of life improves, in-patients have higher demands for basic hospital and nursing care, health education, and psychological support.^26^ According to the study of Jing et al., the current competencies of clinical nurses are still at an intermediate level.^27^ Since the 21st century, there has been a gradual emergence of nurses of various specialties to develop higher nursing skills and possess higher clinical practice, teaching guidance, consultation, and research and information skills.^28^ By further developing the advanced practice roles such as Clinical Nurse Specialist (CNS) developed by the nursing workforce, the professional nursing skills and knowledge they possess will not only enhance the nursing experience and meet the needs of the patient, but also improve the voluntary utilization of health care.^29,30^ According to a study by Leslie et al., advanced practice nurses can promote patient trust and prompt patients to demonstrate good resilience and collaborative skills.^31^ Clinical managers should increase the training of nurses and improve their professional knowledge to assume the role of a health consultant to meet patients’ needs for health and disease-related knowledge.

### Effective Communication with Patients

The results of this study and the other 2 interview-based studies showed that most patients had high requirements for treatment results, especially the patients with incurable malignant diseases.^32,33^ Owing to the continuous reform of the medical system and the gradual improvement of medical technology, patients have excessively high expectations for the treatment effect while seeking medical treatment, which tends to produce discrepancies between expectations and reality, thus, affecting nurse-patient trust.^34^ In addition, with the improvement of patients’ legal consciousness, their requirements for medical service quality and awareness of their medical rights are also constantly increasing. If they are not satisfied with the results, they usually seek legal help, which exacerbates a conflict.^35^ Good communication helps improve patients’ satisfaction and increase trust in nurses.^36^

At the same time, to meet the clinical learning of interns during the epidemic, the interns’ ability for nurse–patient communication should be strengthened. Effective communication strategies are an essential determinant of the positive nurse–patient relationship.^37^ Interns are encouraged to communicate with patients regularly to get patients’ understanding and recognition. The results of Lorena et al. showed that after training, interns could flexibly master communication in conversation methods, and their communication skills improved to a great extent, which is conducive to the coordinated development of the nurse–patient relationship.^38^

### Limitations

For qualitative studies, researchers may be influenced by education and experience, which may result in bias. The interview about the trust in the nurse–patient relationship depends on the patients’ own experiences, and they might have shown a more positive attitude to answer questions when the investigators were nearby. This may become a source of information bias, which should be systematically considered in research. Furthermore, this study was conducted in only 1 hospital. The next step could be to expand hospitals for research and analysis.

## Conclusion

Patients’ trust in nurses not only promotes recovery and motivation, but also goes a long way to maintaining a stable in-patient environment. The multiple factors arising from the outbreak not only affected patients’ trust in nursing staff, but also exposed some of the problems that nurses might have in their work. Under the premise of ensuring medical safety, we can reduce the difficulties of patient admission through a variety of measures such as recruitment, and we can also use the news media to raise patients’ awareness of epidemic prevention and control. While the health care industry is developing at a rapid pace, health care managers should also increase the training of clinical nurses to improve the overall standard of care to help nursing staff better cope with the challenges of the nurse–patient trust relationship in the event of a public health emergency. In addition to this, we should actively communicate with patients to improve their trust in nursing care, which will help us greatly in our work.
